# Interaction between mesenchymal stem cells and endothelial cells restores endothelial permeability via paracrine hepatocyte growth factor *in vitro*

**DOI:** 10.1186/s13287-015-0025-1

**Published:** 2015-03-24

**Authors:** Qi-Hong Chen, Ai-Ran Liu, Hai-Bo Qiu, Yi Yang

**Affiliations:** Department of Critical Care Medicine, Zhong-Da Hospital, School of Medicine, Southeast University, #87 Dingjiaqiao Road, Nanjing, 210009 Jiangsu P.R. China

## Abstract

**Introduction:**

Mesenchymal stem cells (MSCs) have potent stabilising effects on vascular endothelium injury, inhibiting endothelial permeability in lung injury via paracrine hepatocyte growth factor (HGF). Recently, it has been indicated that MSCs secrete more factors by MSC-endothelial cell (MSC-EC) interactions. We hypothesised that MSC-EC interactions restore endothelial permeability induced by lipopolysaccharide (LPS) via paracrine HGF.

**Methods:**

We investigated the endothelial permeability induced by LPS under two co-culture conditions. Human pulmonary microvascular endothelial cells (HPMECs) were added into the upper chambers of cell-culture inserts, while two different co-culture conditions were used in the lower side of the transwells, as follows: (1) MSC-EC interaction group: MSCs and HPMECs contact co-culture; (2) MSC group: MSCs only. The endothelial paracellular and transcellular permeabilities in the upper side of transwells were detected. Then the concentration of HGF was measured in the culture medium by using an enzyme-linked immunosorbent assay kit, followed by neutralisation of HGF with anti-HGF antibody in the co-culture medium. In addition, adherens junction and cytoskeleton protein expressions were measured by Western blot and immunofluorescence. HPMEC proliferation was analysed by bromodeoxyuridine incorporation assay.

**Results:**

The paracellular permeability significantly increased after LPS stimulation in a dose-dependent and time-dependent manner. Meanwhile, MSC-EC interaction more significantly decreased endothelial paracellular and transcellular permeability induced by LPS. Moreover, HGF levels in the MSC-EC interaction group were much higher than those of the MSC group. However, neutralising HGF with anti-HGF antibody inhibited the role of MSC-EC interaction in improving endothelial permeability. Compared with the MSC group, MSC-EC interaction increased vascular endothelial (VE)-cadherin and occludin protein expression, reduced caveolin-1 protein expression in HPMECs, and restored remodelling of F-actin and junctional localisation of VE-cadherin. Furthermore, the proliferation ratio in the MSC-EC interaction group was higher than that of the MSC group. However, the effects of MSCs were significantly blocked by anti-HGF antibody.

**Conclusions:**

These data suggested that MSC-EC interaction decreased endothelial permeability induced by LPS, which was attributed mainly to HGF secreted by MSCs. The main mechanisms by which HGF restored the integrity of endothelial monolayers were remodelling of endothelial intercellular junctions, decreasing caveolin-1 protein expression, and inducing proliferation in HPMECs.

**Electronic supplementary material:**

The online version of this article (doi:10.1186/s13287-015-0025-1) contains supplementary material, which is available to authorized users.

## Introduction

Acute lung injury (ALI) involves a disruption of the alveolar-capillary membranes, with an excessive and uncontrolled inflammatory response leading to pulmonary oedema with serum proteins and oedema fluid [1]. ALI pathogenesis is still only partly understood; however, pulmonary endothelial cell (EC) dysfunction is a key component of ALI pathogenesis because EC play a major role by changing their barrier permeability [2]. As ALI is characterised by endothelial hyperpermeability, stabilising EC barrier function is critical for treating ALI [3].

A growing number of studies have provided convincing data on the beneficial effects of mesenchymal stem cells (MSCs) in treating ALI induced by endotoxin [4-6]. Studies have shown that MSCs have potent stabilising effects on vascular endothelium injury by inhibiting endothelial permeability after injury via modulation of adherens junction (AJ) proteins [7]. However, the detailed pathogenesis of MSCs in improving endothelial injury is still unclear. Much of the current research has suggested that multipotent differentiation of MSCs contributes minimally to the beneficial effects but that paracrine activity plays a predominant role [8,9]. Thus, MSCs improve endothelial injury mainly through a paracrine mechanism.

Hepatocyte growth factor (HGF) is a multifunctional, mesenchyme-derived pleiotropic factor secreted by MSCs [10-12]. HGF appears in lung circulation under pathological conditions, such as ALI, and exhibits sustained barrier-protective effects on human pulmonary ECs [13]. MSCs secrete a small amount of HGF under normal conditions; however, high HGF levels have been detected in MSC medium under pathological conditions [14-16]. Recently, it has been found that MSCs secrete more factors following MSC-EC interactions [17]. Therefore, HGF resulting from MSC-EC interactions could be the key factor from MSCs that improve endothelial permeability.

The aim of the present study was to illuminate the effect and mechanism of MSC-EC interaction in the integrity of an EC monolayer induced by lipopolysaccharide (LPS). We investigated the effect of MSC-EC interaction on endothelial paracellular and transcellular permeability by performing two *in vitro* co-culture experiments and then explored the role and mechanism of HGF in regulating the integrity of a human pulmonary microvascular EC (HPMEC) monolayer by neutralising HGF with HGF antibody.

## Methods

### Human mesenchymal stem cell culture

**Figure 1:**
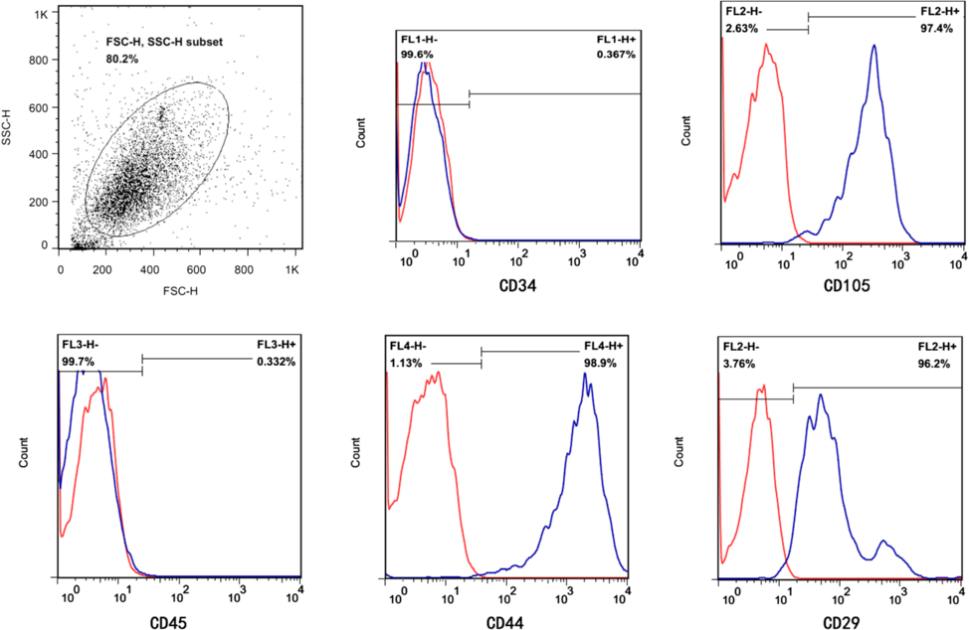
**Flow cytometry identification of human mesenchymal stem cells (hMSCs).** Cell surface markers of hMSCs, including CD34, CD44, CD105, CD29, and CD45, were analysed with flow cytometry. Red lines represent the isotype controls. The supplier guarantees that there is no ethical issue in using the hMSCs for experiments. FSC-H, forward scatter height; SSC-H, side scatter height.

**Figure 2:**
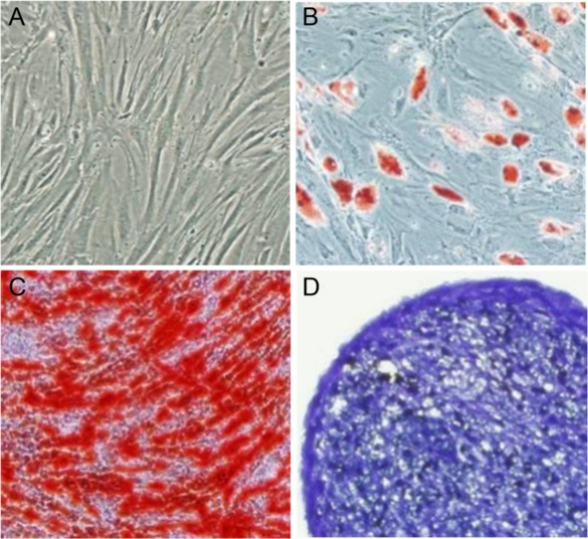
**Multilineage differentiation identification of human mesenchymal stem cells.** Morphology of human mesenchymal stem cells at the third passage **(A)** (100 T) and multilineage differentiation capacities of mouse mesenchymal stem cells, including adipogenic differentiation stained with Oil Red O **(B)** (200 T), osteogenic differentiation stained with Alizarin red **(C)** (200 T), and chondrogenic differentiation stained with Toluidine blue **(D)** (200 T), were observed by microscope.

Human mesenchymal stem cells (hMSCs) and HPMECs were used in the present study. hMSCs were purchased from Cyagen Biosciences Inc. (Guangzhou, China). An additional statement of ethics for hMSC use shows this in more detail (Additional file 1). The cells were identified by detecting cell surface phenotypes. Fluorescein-conjugated monoclonal antibodies, including CD29, CD34, CD44, CD105, and CD45, and the respective isotype controls were purchased from Miltenyi Biotec (Bergisch Gladbach, Germany). Flow cytometry was performed with fluorescence-activated cell sorting analysis (Figure 1). The multipotent potential for differentiation along adipogenic, osteogenic, and chondrogenic lineages was determined by staining with Oil Red O, Alizarin red, or Toluidine blue, respectively, followed by culture in adipogenic, osteogenic, or chondrogenic differentiation media (Cyagen Biosciences Inc.) for 2 to 3 weeks (Figure 2), thus verifying their identity as hMSCs. hMSCs were cultured in MSC growth medium (Cyagen Biosciences Inc.). All cells were cultured in a humidified 5% CO_2_ incubator at 37°C. The culture media were changed every 3 days, and the cells were used at passages 3 to 7 for all experiments.

### Human pulmonary microvascular endothelial cell culture

First-passage HPMECs were obtained from ScienCell Research Laboratories (Carlsbad, CA, USA). An additional ethical approval for HPMEC use shows this in more detail (Additional file 2). HPMECs were routinely characterised by the supplier. HPMECs were added into the upper chambers of 0.4-μm cell-culture inserts at a density of 50,000 cells per insert well and cultured in EGM-2 (endothelial growth media-2) (ScienCell Research Laboratories). The culture media were changed every 3 days, and the cells were used at passages 3 to 7 for all experiments.

### Mesenchymal stem cell-endothelial cell interaction through transwell inserts

HPMECs were added to the upper chambers of 0.4-μm cell-culture inserts (0.4-μm pore size polyester membrane from Corning Inc., Corning, NY, USA) at a density of 50,000 cells per insert well. Different co-culture conditions were used in the lower side of transwells: (1) MSC-EC interaction group: hMSCs mixed with HPMECs were plated in the lower chambers at a density of 100,000 cells/cm^2^; (2) MSC group: Only MSCs were plated in the lower chambers. After growing to confluence, the medium was changed with Dulbecco’s modified Eagle’s medium/F12 supplemented with 10% fetal bovine serum (Wisent Inc., Nanjing, China) containing 0, 1, 10, 100, 1,000, and 10,000 ng/mL LPS (Sigma-Aldrich, St. Louis, MO, USA) and cultured for 0, 1, 2, 6, 12, and 24 hours, respectively.

### Human pulmonary microvascular endothelial cell permeability examination

HPMECs were seeded at 50,000 cells per insert well and cultured for 1 to 3 days to allow the growth of a confluent monolayer. Monolayers were treated with 100 ng/mL LPS for 6 hours before testing permeability. Paracellular permeability was tested by adding 10 μL of 10 mg/mL fluorescein isothiocyanate (FITC)-Dextran (Sigma-Aldrich) to the upper chamber, as previously described [18]. The FITC component from samples was obtained 40 minutes after the addition of FITC-Dextran. Medium (100 μL) was withdrawn from the lower well and upper well, respectively. Measurements were taken with a microplate reader by using excitation and emission wavelengths of 490 and 525 nm, respectively. Permeability was calculated as previously described [19]. To measure transcellular permeability, 10 μL of 0.4 mg/mL FITC-bovine serum albumin (FITC-BSA) (Invitrogen, Waltham, MA, USA) was added to the upper chamber. The remaining experimental procedure of transcellular permeability detection is the same as that of paracelluar permeability.

### Enzyme-linked immunosorbent assay

hMSCs co-cultured with HPMECs in different co-culture conditions were stimulated by LPS. After 6 hours of treatment, supernatants were collected and centrifuged to remove debris. HGF was determined via an enzyme-linked immunosorbent assay (ELISA) by using commercially available ELISA sets (ExCell Biology, Inc., Shanghai, China). To remove the effect of cell numbers on HGF secretion, we further tested HGF concentration by ELISA quantification in conditioned medium from six different formats: HPMEC alone, MSC alone, the sum of HPMEC and MSC alone, the upper of MSC-EC indirect co-culture, the lower of MSC-EC indirect co-culture, and MSC-EC direct co-culture. In each format, the number of cells seeded in the culture was held constant at 1 × 10^5^ and cultured for 1 to 3 days to allow the growth of a confluent monolayer. Monolayers were treated with 100 ng/mL LPS for 6 hours before examination. ELISA was performed in accordance with the instructions of the manufacturer. All samples were measured in duplicate.

### Immunofluorescence

In total, 5 × 10^4^ HPMECs were seeded on the upper chambers of 0.4-μm cell-culture inserts and cultured for 1 to 3 days to allow the growth of a confluent monolayer. Monolayers were treated with 100 ng/mL LPS before immunofluorescence. After 6-hour treatment, cells were washed in cold phosphate-buffered saline (PBS) and fixed in 4% paraformaldehyde. Non-specific binding of antibody was blocked in 1% BSA in PBS for 30 minutes. Cells were stained with vascular endothelial (VE)-cadherin antibody (Cell Signaling Technology, Danvers, MA, USA) or F-actin antibody (Abcam, Hong Kong) at 4°C overnight and then washed with PBS. FITC-conjugated goat anti-mouse IgG (Jackson ImmunoResearch Laboratories, Inc., West Grove, PA, USA) was used as a secondary antibody. Finally, DAPI (4,6-diamidino-2-phenylindole) was used to stain nuclei. Samples were examined with a microscope (Olympus, Tokyo, Japan) equipped with fluorescent illumination.

### Western blot analysis

Total protein from cells was extracted by using RIPA lysis buffer (Beyotime Institute of Biotechnology, Shanghai, China) supplemented with 1 mmol/L phenylmethylsulfonyl fluoride (PMSF), followed by separation by 6% or 12% sodium dodecyl sulphate-polyacrylamide gel electrophoresis and transfer onto PVDF (polyvinylidene fluoride) membranes (Well-Offer, Nanjing, China). Membranes were then blocked in PBS-T containing 5% milk for 2 hours at room temperature and incubated at 4°C overnight with primary antibodies against VE-cadherin (Cell Signaling Technology) or caveolin-1 (Epitomics, Burlingame, CA, USA). On the following day, blots were washed in PBS-T and incubated in peroxidase-conjugated secondary antibody (HuaAn Biotechnology, Hangzhou, China) for 1 hour at room temperature. Immunoreactive bands were obtained by using a chemiluminescence imaging system (ChemiQ 4800mini; Ouxiang, Shanghai, China) after incubation with horseradish peroxidase.

### Human pulmonary microvascular endothelial cell proliferation

To explore the role of HGF secreted by MSCs in HPMEC proliferation, cell proliferation was analysed by bromodeoxyuridine (BrdU) incorporation assay by using a FITC-conjugated Brdu flow kit as described by the manufacturer (KeyGEN bioTECH, Nanjing, China). Briefly, HPMECs were seeded at 50,000 cells per insert well and cultured for 1 to 3 days to allow the growth of a confluent monolayer. MSCs co-cultured with HPMECs in different co-culture conditions were stimulated by LPS. Before termination of experiments, HPMECs were incubated with 10 μM BrdU for 6 hours before analysis.

### Statistical analysis

The results were presented as the mean ± standard deviation. Statistical analyses were performed by using the SPSS 16.0 software package (SPSS Inc., Chicago, IL, USA). For group comparison, a one-way analysis of variance was used, followed by Tukey’s test. A *P* value less than 0.05 was considered statistically significant.

## Results

### Lipopolysaccharide evoked human pulmonary microvascular endothelial cell hyperpermeability

**Figure 3:**
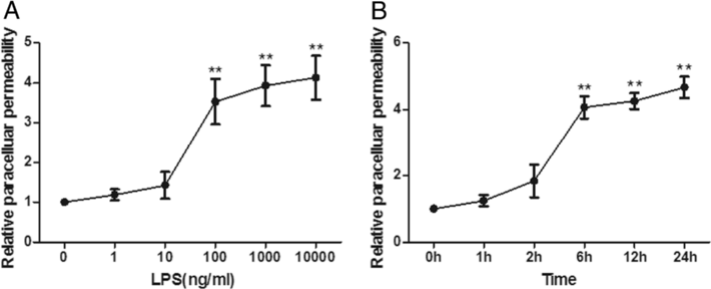
**Lipopolysaccharide (LPS) evokes human pulmonary microvascular endothelial cell hyperpermeability.** The paracellular permeability of human pulmonary microvascular endothelial cells was measured at different LPS dosages and at different time points after LPS intervention. The results showed that paracellular permeability had no significant alterations after LPS addition with dosages below 10 ng/mL. However, the paracellular permeability significantly increased after LPS stimulation with dosages above 100 ng/mL in a dose-dependent manner **(A)**. Furthermore, we observed that paracellular permeability significantly increased after 6 hours following 100 ng/mL LPS addition in a time-dependent manner **(B)**. Shown are the mean ± standard error of three parallel experiments (n = 3, **P <0.01 versus group 0 ng/mL or 0 hours).

LPS was used to induce endothelial injury and increase endothelial permeability in our study. To find the optimum dosage and time for LPS stimulation, we measured the paracellular permeability of HPMECs in response to different LPS dosages at different time points after LPS intervention. The results showed that the paracellular permeability had no significant alterations after LPS addition with a dosage below 10 ng/mL. However, the paracellular permeability significantly increased after LPS stimulation with a dosage above 100 ng/mL in a dose-dependent manner (*P* <0.01) (Figure 3A). Furthermore, we observed that the paracellular permeability significantly increased after 6 hours following 100 ng/mL LPS addition in a time-dependent manner (*P* <0.01) (Figure 3B). Therefore, we studied HPMECs at 6-hour exposure to 100 ng/mL LPS in subsequent experiments.

### Mesenchymal stem cell-endothelial cell interaction restored the integrity of human pulmonary microvascular endothelial cell monolayers

**Figure 4:**
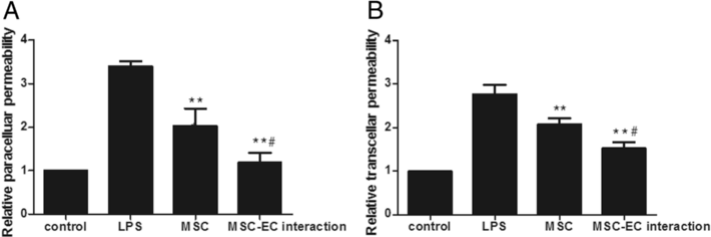
**Mesenchymal stem cell-endothelial cell (MSC-EC) interaction promoted human MSCs to restore the integrity of human pulmonary microvascular endothelial cell (HPMEC) monolayers.** We found HPMEC paracellular permeability for both MSC-EC interaction and MSC groups significantly decreased (*P* <0.01) **(A)**. In addition, HPMEC paracellular permeability in the MSC-EC interaction group decreased more significantly than that of the MSC group (*P* <0.05) (A). Similarly, HPMEC transcellular permeability significantly decreased in two co-culture systems (*P* <0.01) **(B)**, while MSC-EC interaction more significantly decreased HPMEC transcellular permeability (*P* <0.05) (B). (n=3, ** p < 0.01 vs. group LPS; # p < 0.05 vs. group MSC). LPS, lipopolysaccharide.

Microenvironment plays important roles in MSC biological function, through either direct or indirect contact by paracrine-soluble factors from the neighbouring cells [20]. To evaluate the effect of MSC-EC contact on HPMEC permeability, we introduced two co-culture systems in our study and compared the paracellular and transcellular permeability under these conditions. We found that HPMEC paracellular permeability was significantly decreased by both MSC-EC interaction and the MSC group (*P* <0.01; Figure 4A). In addition, HPMEC paracellular permeability was more significantly decreased by the MSC-EC interaction group than the MSC group (*P* <0.05; Figure 4A). Similarly, HPMEC transcellular permeability significantly decreased in both co-culture systems (*P* <0.01; Figure 4B), while MSC-EC interaction more significantly decreased HPMEC transcellular permeability (*P* <0.05; Figure 4B).

### More hepatocyte growth factor was secreted under mesenchymal stem cell-endothelial cell interactions

**Figure 5:**
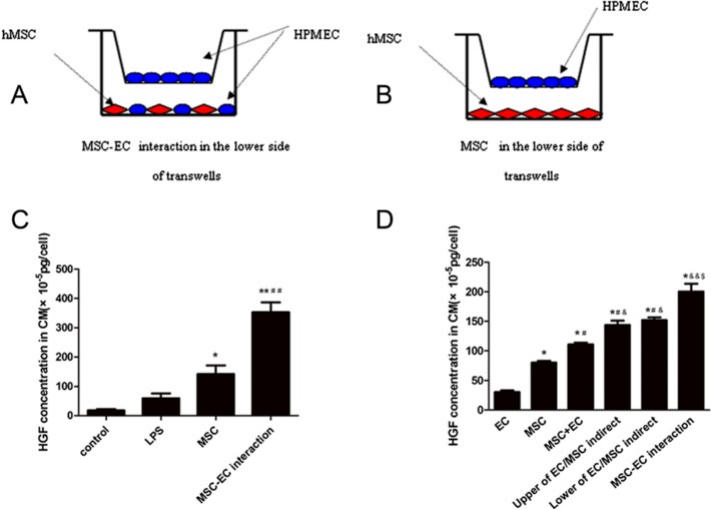
**More hepatocyte growth factor (HGF) was secreted under mesenchymal stem cell-endothelial cell (MSC-EC) interactions.** We adopted two different co-culture conditions through transwell insert including MSC-EC interactions **(A)** and MSCs **(B)**. Then we measured the concentration of HGF in the culture medium by using an enzyme-linked immunosorbent assay (ELISA) kit. The results indicated that the concentration of HGF in direct co-culture and indirect co-culture media was significantly higher than that of the lipopolysaccharide (LPS) group. Interestingly, HGF levels in direct co-culture medium were much higher than that of indirect co-culture medium **(C)** (n = 3, **P <0.01 versus LPS group; ^##^ *P* <0.01 versus indirect co-culture group). We further tested HGF concentration by ELISA quantification in conditioned medium from six different formats. The results showed that the concentration of HGF in the MSC group was significantly higher than that of the EC group. HGF levels had no significant difference between the upper and lower of the MSC-EC indirect co-culture group, which were higher compared with the MSC group and the sum of the human pulmonary microvascular endothelial cell (HPMEC) and MSC alone group **(D)** (*P* <0.05 and *P* <0.01). However, HGF levels in the MSC-EC interaction group were highest in all six groups (D) (*P* <0.01) (n = 3, *P <0.01 versus EC group, ^#^ *P* <0.01 versus MSC group; ^&^ *P* <0.05, ^&&^ *P* <0.01 versus MSC + EC group, ^$^ *P* <0.01 versus upper of MSC-EC indirect group or lower of MSC-EC indirect group). CM, conditioned media; hMSC, human mesenchymal stem cell.

We measured the concentration of HGF in the culture medium by using an ELISA kit. Interestingly, the results indicated that the concentration of HGF in the MSC-EC interaction group and the MSC group was significantly higher than that of the LPS group. HGF levels in the MSC-EC interaction group were much higher than that of the MSC group (*P* <0.01; Figure 5C). These data suggested that hMSCs might secrete more HGF by direct culture with HPMECs than MSCs only directly cultured with HPMECs. To remove the effect of cell numbers on HGF secretion, we further tested HGF concentration by ELISA quantification in conditioned medium from six different formats: HPMEC alone, MSC alone, the sum of HPMEC and MSC alone, the upper of MSC-EC indirect co-culture, the lower of MSC-EC indirect co-culture, and MSC-EC direct co-culture. The results showed that the concentration of HGF in the MSC group was significantly higher than that of the EC group. HGF levels had no significant difference between the upper and lower of MSC-EC indirect co-culture group, which were higher compared with the MSC group and the sum of HPMEC and MSC-alone group (*P* <0.05 and *P* <0.01; Figure 5D). However, HGF levels in the MSC-EC interaction group were highest in all six groups (*P* <0.01; Figure 5D).

### Hepatocyte growth factor neutralisation attenuated the restoration of human pulmonary microvascular endothelial cell monolayer integrity in mesenchymal stem cell-endothelial cell interaction conditions

**Figure 6:**
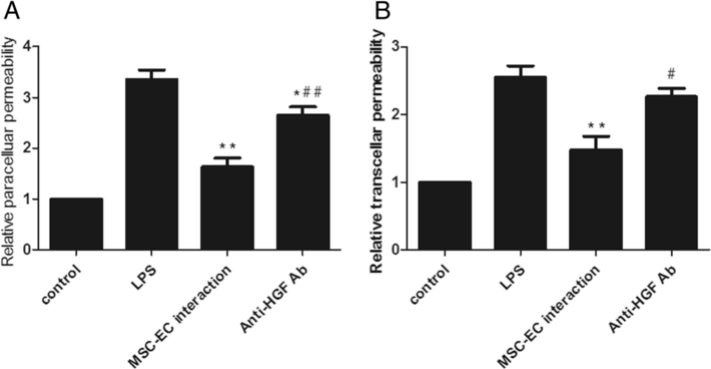
**Hepatocyte growth factor (HGF) neutralisation attenuated the restoration of the integrity of human pulmonary microvascular endothelial cell (HPMEC) monolayers in mesenchymal stem cell-endothelial cell (MSC-EC) interaction conditions.** We found that the paracellular and transcellular permeability in the anti-HGF antibody (Ab) group was significantly increased over control (without anti-HGF Ab) (n = 3, *P* <0.05) **(A, B)**, suggesting that human MSCs restored the integrity of HPMEC monolayers in MSC-EC interaction by paracrine HGF. (n=3, ** p < 0.01 vs. group LPS; # p<0.05 # # p < 0.01 vs. group MSC-EC interaction) LPS, lipopolysaccharide.

As HGF levels were upregulated in parallel to restore the integrity of HPMEC monolayers in MSC-EC interaction conditions, we further investigated the role of HGF in regulating the integrity of HPMEC monolayers. hMSCs were co-cultured with direct contact to HPMECs in the presence of 100 ng/mL anti-HGF antibody (Abcam) for 24 hours. We found that the paracellular and transcellular permeability in the anti-HGF antibody group was significantly increased over the control (without anti-HGF antibody) (n = 3, *P* <0.05) (Figure 6A, B), suggesting that hMSCs restored the integrity of HPMEC monolayers in MSC-EC interaction conditions by paracrine HGF.

### Hepatocyte growth factor secreted in mesenchymal stem cell-endothelial cell interaction conditions upregulated endothelial vascular endothelial-cadherin protein and decreased caveolin-1 protein expression

**Figure 7:**
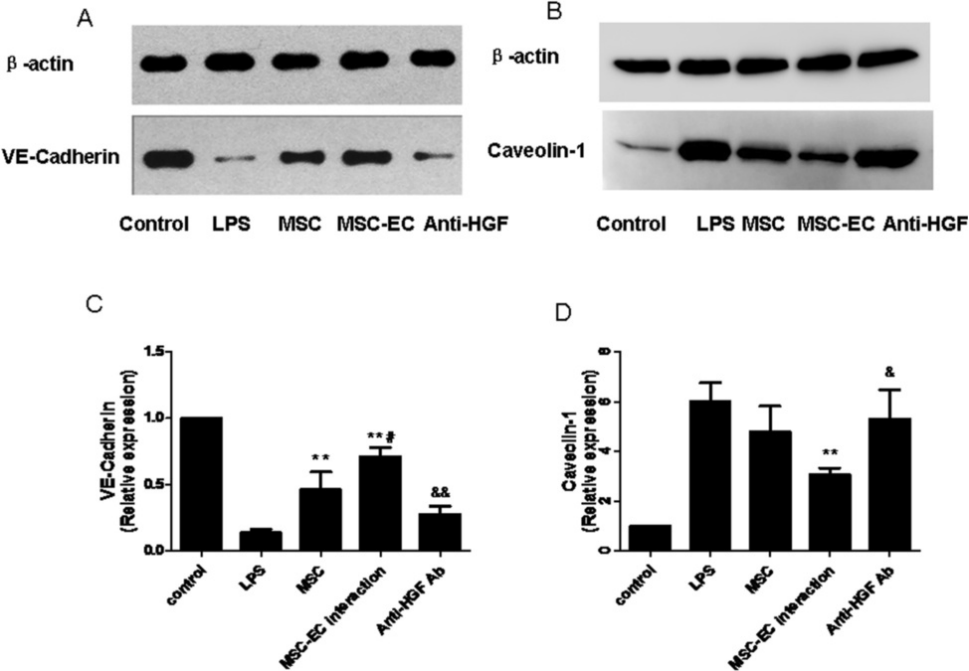
**Hepatocyte growth factor (HGF) secreted by human mesenchymal stem cells (hMSCs) following mesenchymal stem cell-endothelial cell (MSC-EC) interactions upregulated vascular endothelial (VE)-cadherin protein and decreased caveolin-1 protein expression.** Compared with the MSC group, MSC-EC interaction increased VE-cadherin protein expression (*P* <0.01) **(A, C)** while reducing caveolin-1 protein expression in human pulmonary microvascular endothelial cells (HPMECs) (*P* <0.01) **(B, D)**. However, the MSC effect was significantly blocked by anti-HGF antibody (*P* <0.05 or *P* <0.01) (C, D). These results indicated that HGF secreted by hMSCs upregulated endothelial VE-cadherin protein and decreased caveolin-1 protein expression. (n=3, * *p < 0.01 vs. group LPS, # p < 0.01 vs. MSC; & p< 0.05 && p < 0.01 vs. group MSC-EC interaction) LPS, lipopolysaccharide.

AJs represent the majority of junctions comprising the endothelial paracellular barrier. AJs are composed of VE-cadherin [21], whereas caveolin-1 is found in small invaginations in the cell membrane and is thought to play an important role in regulating the transcellular permeability of HPMECs [22]. Therefore, in the present study, to explore the mechanism of how HGF secreted by MSCs improves endothelial barrier function, we examined endothelial VE-cadherin and caveolin-1 protein expression under the co-culture conditions. Compared with the MSC group, MSC-EC interaction increased VE-cadherin protein expression (*P* <0.01; Figure 7A, C) while reducing caveolin-1 protein expression in HPMECs (*P* <0.01; Figure 7B, D). However, the MSC effect was significantly blocked by anti-HGF antibody (*P* <0.05 or *P* <0.01; Figure 7C, D). These results indicated that HGF secreted by the MSC-EC interaction upregulated endothelial VE-cadherin protein and decreased caveolin-1 protein expression.

### Hepatocyte growth factor secreted in mesenchymal stem cell-endothelial cell interaction conditions restored remodelling of endothelial F-actin and vascular endothelial-cadherin

**Figure 8:**
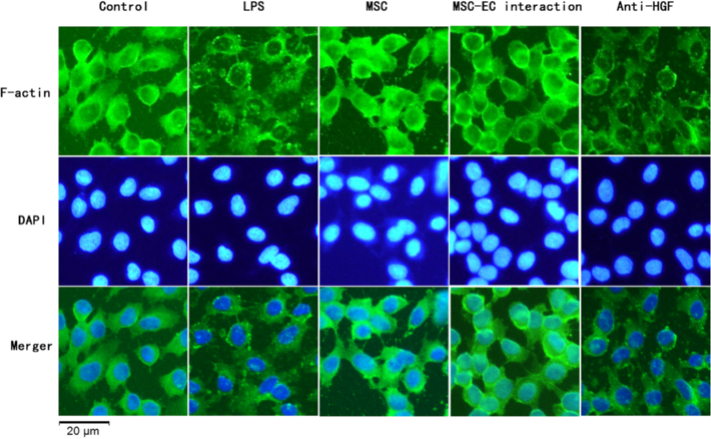
**Hepatocyte growth factor (HGF) secreted in mesenchymal stem cell-endothelial cell (MSC-EC) interactions conditions restored remodelling of endothelial F-actin.** The results indicated that human pulmonary microvascular endothelial cells cultured under normal condition had F-actin localised mainly at the cell periphery. Lipopolysaccharide (LPS) induced dispersion of F-actin. After 7 days of direct or indirect co-culture with human MSCs, remodelling of F-actin was partially restored. However, neutralising HGF from conditioned media with anti-HGF antibody caused F-actin to be disrupted again. DAPI, 4,6-diamidino-2-phenylindole.

**Figure 9:**
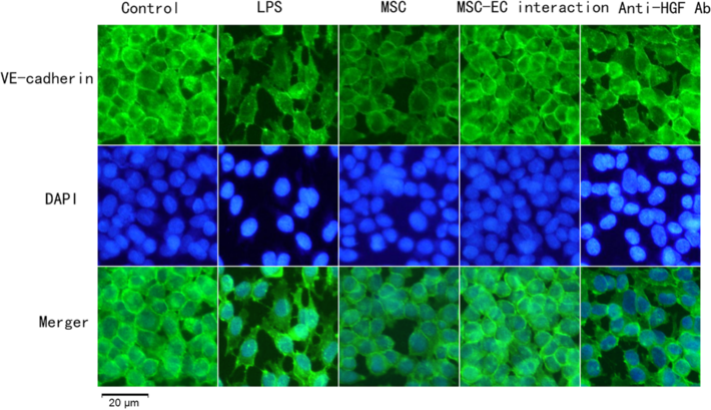
**Hepatocyte growth factor (HGF) was secreted in mesenchymal stem cell-endothelial cell (MSC-EC) interaction conditions and restored remodelling of vascular endothelial (VE)-cadherin.** The results indicated that human pulmonary microvascular endothelial cells cultured under normal conditions had VE-cadherin, the main component of adherens junctions, forming intercellular junctions. Lipopolysaccharide (LPS) induced dispersion of VE-cadherin. After 7 days of direct or indirect co-cultured with human MSCs, VE-cadherin was partially restored. However, neutralising HGF from conditioned media with anti-HGF antibody (Ab) caused VE-cadherin to be disrupted again. DAPI, 4,6-diamidino-2-phenylindole.

We further investigated the role and mechanism of MSC-EC interaction in regulating remodelling of the endothelial actin cytoskeleton and intercellular AJs by immunofluorescence. The results indicated that HPMECs cultured under normal conditions had F-actin localised mainly at the cell periphery but that VE-cadherin, the main component of AJs, formed intercellular junctions. LPS induced dispersion of F-actin and VE-cadherin. After 3 to 5 days of MSC-EC interaction co-culture, remodelling of F-actin and junctional localisation of VE-cadherin was partially restored. However, neutralising HGF from the conditioned media with anti-HGF antibody caused F-actin (Figure 8) and VE-cadherin (Figure 9) to again be disrupted. These data suggested that HGF secreted following MSC-EC interaction restored remodelling of endothelial F-actin and VE-cadherin.

### Hepatocyte growth factor secreted by mesenchymal stem cell-endothelial cell interaction induces proliferation in human pulmonary microvascular endothelial cells

**Figure 10:**
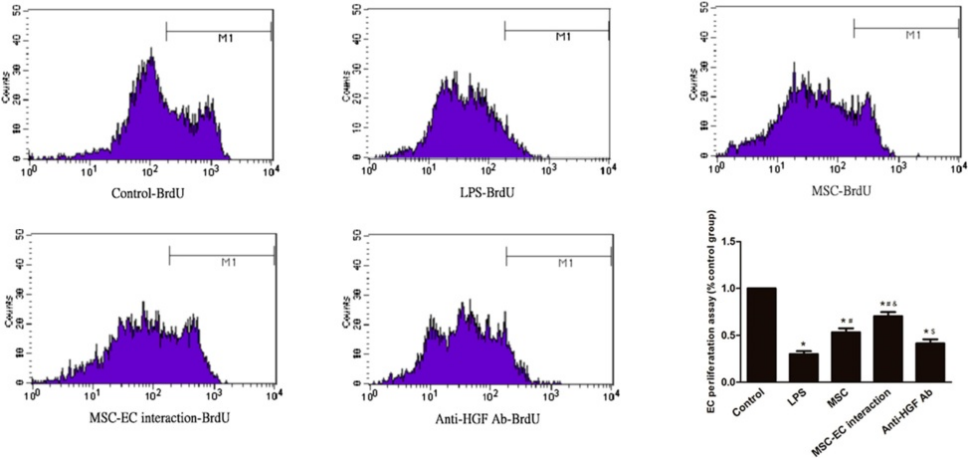
**Hepatocyte growth factor (HGF) secreted by mesenchymal stem cell-endothelial cell (MSC-EC) interaction induces proliferation in human pulmonary microvascular endothelial cells (HPMECs).** To explicit effect of HGF secreted by MSCs on HPMEC proliferation, cell proliferation was analysed by bromodeoxyuridine (BrdU) incorporation assay. The results indicated that MSCs induce proliferation in HPMECs injured by lipopolysaccharide (LPS). Interestingly, the proliferation ratio in the MSC-EC interaction group was higher than that of the MSC group (*P* <0.05). However, the MSC-EC interaction effect was significantly blocked by anti-HGF antibody (*P* <0.01), which explains why there are more HPMECs in the MSC-EC group. The result suggested that HGF secreted by MSC-EC interaction induces proliferation in HPMECs (n = 3, *P <0.01 versus control group, ^#^ *P* <0.01 versus LPS group; ^&^ *P* <0.05 versus MSC group, ^$^ *P* <0.01 versus MSC-EC interaction).

To explore the role of HGF secreted by MSCs in HPMEC proliferation, cell proliferation was analysed by BrdU incorporation assay as described in Xiong G *et al*. [23]. The results indicated that MSCs induce proliferation in HPMECs injured by LPS; interestingly, the proliferation ratio in the MSC-EC interaction group was higher than that of the MSC group (*P* <0.05; Figure 10); however, the MSC-EC interaction effect was significantly blocked by anti-HGF antibody (*P* <0.01; Figure 10), which explains why there are more HPMECs in the MSC-EC group. The result suggested that HGF secreted by MSC-EC interaction induces proliferation in HPMECs.

## Discussion

ALI involves a disruption of the integrity of the endothelial barrier, which causes pulmonary oedema with serum proteins and oedema fluid. MSCs appear to restore endothelial function, via both paracrine and cell contact-dependent effects [17,24]. However, the detailed mechanism of how MSCs improve endothelial injury is still unclear. In the present study, we found that MSC-EC interaction promotes MSCs to restore the integrity of EC monolayers induced by LPS via paracrine HGF, which restore remodelling of endothelial intercellular AJs, decrease caveolin-1 protein expression, and induce proliferation in HPMECs.

Pulmonary endothelium serves as a semi-selective barrier between the plasma and interstitium to micromolecules and bioactive agents. Endothelial injury results in barrier dysfunction, which contributes to pulmonary oedema in lung injury [1]. There are two pathways regulating permeability across the endothelial barrier: paracellular and transcellular [25]. Paracellular permeability is determined primarily by AJs and tight junctions. In contrast, the transcellular pathway is defined as vesicle-mediated transport of macromolecules, such as albumin, across the endothelial barrier in a caveolae-dependent manner [22]. Endotoxin, the bacterial LPS, is considered an important factor in the development of ALI, which increases the pulmonary endothelial paracellular and transcellular permeability that causes pulmonary oedema [3]. LPS has been widely adopted for *in vivo* and *in vitro* studies of endothelial permeability injury mode [26-28]. In this study, we used an EC permeability injury model induced by LPS, which caused an increase in EC paracellular permeability in a time- and dose-dependent manner. Our study showed that the paracellular permeability significantly increased after 6 hours following 100 ng/mL LPS addition, in accordance with the previous study [29]. Therefore, we studied HPMECs at 6-hour exposure to 100 ng/mL LPS in subsequent experiments.

MSCs have potent stabilising effects on vascular endothelium injury by inhibiting endothelial permeability. A strong paracrine capacity has been proposed as the principal mechanism [7]. MSCs are capable of secreting a few factors under normal conditions. Thus, some studies make an effort to maximise the paracrine potential of MSCs. For instance, hypoxic preconditioning of MSCs facilitated release of trophic factors [14]. Furthermore, Pati S *et al*. showed that conditioned medium from MSC-EC co-cultures, but not from ECs or MSCs cultured alone, can decrease endothelial permeability [18], suggesting that MSCs can exert a better effect through the secretion of soluble factors induced by contact with ECs [30][31]. To clarify the role of paracrine interactions between ECs and MSCs, we adopted an MSC-EC interaction co-culture system in our study. Our data demonstrated that MSC-EC interaction restores the integrity of HPMEC monolayers. These findings suggest that MSCs in contact with ECs can exert a beneficial effect, possibly through the increased secretion of a soluble factor.

HGF is a multifunctional, mesenchyme-derived pleiotropic factor secreted by several cell types [32-34] and regulates many biological events, such as cell mitogenesis, organogenesis, morphogenesis, and cell survival [35,36]. Novel therapeutic strategies using HGF to fight lung diseases have been suggested because HGF exhibited sustained barrier-protective effects on ECs [34]. Recently, it has been suggested that HGF appears in lung circulation under ALI, exhibiting sustained barrier-protective effects on human pulmonary ECs [34]. HGF resulting from MSC-EC interaction may be responsible for improving HPMEC permeability. Our results indicated that MSCs or HPMECs alone secrete little HGF but that MSCs in contact with ECs secrete more HGF in parallel with restoring the integrity of HPMEC monolayers. However, the effect of MSCs was significantly blocked following HGF neutralisation with anti-HGF antibody. Thus, HGF, a soluble factor secreted by hMSCs, may account for the effect that restored the integrity of EC monolayers in direct co-culture conditions.

HGF-induced endothelial permeability effects on ECs have been associated with remodelling of the actin cytoskeleton as well as the increased expression of the AJ protein VE-cadherin. Endothelial paracellular permeability is determined primarily by AJs and tight junctions [21]. ECs express high levels of VE-cadherin, which interacts on neighbouring ECs to form AJs. AJs represent the majority of junctions comprising the endothelial paracellular barrier. AJs are composed of VE-cadherin [37]. Our results showed that LPS stimulation caused dispersion of VE-cadherin but that MSC-EC interaction increased VE-cadherin protein expression and restored remodelling of endothelial F-actin, which restored the integrity of EC monolayers. However, the MSC effect was significantly blocked by anti-HGF antibody. Thus, HGF secreted by MSCs following MSC-EC interaction restored paracellular permeability by remodelling of endothelial F-actin and VE-cadherin.

Caveolin-1 is thought to play an important role in regulating endothelial transcellular permeability [19,38]. The transcellular pathway is defined as vesicle-mediated transport of macromolecules, such as albumin, across the endothelial barrier in a caveolae-dependent manner. Caveolin-1 regulates endothelial transcellular transport of macromolecules [22] that, in turn, is concomitant with the onset of the increase of transcellular permeability. LPS was shown to induce the expression of caveolin-1 in ECs and thereby contribute to the mechanism of increased transcellular permeability [39,40]. Similarly, our data showed that MSCs co-cultured in contact with ECs more significantly decreased caveolin-1 protein expression in ECs. However, the effect of MSCs was significantly blocked by anti-HGF antibody. These results indicated that HGF secreted by MSCs following MSC-EC interaction restores transcellular permeability by reducing caveolin-1 protein expression.

A limitation of our study should be noted. Our study suggested that HGF secreted by hMSCs following MSC-EC interaction may account for the effect that restored the integrity of EC monolayers. However, ECs can also secrete a small amount of HGF induced by LPS. Thus, a small amount of HGF secreted following MSC-EC interaction comes from ECs. To solve this problem, the identification of HGF protein expression in MSCs may be helpful. However, it is very difficult to separate MSCs from ECs after MSC-EC contact during co-culture. Our future studies will employ techniques such as magnetic cell sorting to separate MSCs from ECs.

## Conclusions

In summary, here we have shown that MSC-EC interaction decreased endothelial paracellular and transcellular permeability induced by LPS. HGF secreted by hMSCs may account for the effect that restored the integrity of EC monolayers. The main mechanisms by which HGF restored paracellular permeability are remodelling of endothelial intercellular AJs, decreasing caveolin-1 protein expression, and inducing proliferation in HPMECs.

## Additional files

Additional file 1:
**A statement of ethics for human mesenchymal stem cell (hMSC) use.** A statement of ethics was used to guarantee there is no ethical issue in using the hMSCs for experiments. Additional file 2:
**Ethical approval for human pulmonary microvascular endothelial cell (HPMEC) use.** An ethical approval was provided to guarantee there was no ethical issue in using the HPMECs for experiments.

## Abbreviations

AJ: adherens junction
ALI: acute lung injury
BrdU: bromodeoxyuridine
BSA: bovine serum albumin
EC: endothelial cell
ELISA: enzyme-linked immunosorbent assay
FITC: fluorescein isothiocyanate
HGF: hepatocyte growth factor
hMSC: human mesenchymal stem cell
HPMEC: human pulmonary microvascular endothelial cell
LPS: lipopolysaccharide
MSC: mesenchymal stem cell
MSC-EC: mesenchymal stem cell-endothelial cell
PBS: phosphate-buffered saline
VE: vascular endothelial
