# Supplementary material for: Interaction between mesenchymal stem cells and endothelial cells restores endothelial permeability via paracrine hepatocyte growth factor in vitro
Source: Stem Cell Res Ther. 2015 Mar 24;6(1):44. doi: 10.1186/s13287-015-0025-1 (PMC4431320; doi:10.1186/s13287-015-0025-1)
Supplement: Additional file 1: — A statement of ethics for human mesenchymal stem cell (hMSC) use. A statement of ethics was used to guarantee there is no ethical issue in using the hMSCs for experiments. [file 13287_2015_25_MOESM1_ESM.pdf]

Ethical Standards Resources

For over a decade, ScienCell Research Laboratories has been a leading provider of human and animal primary cells, cell-derived DNA, RNA and protein, as well as other biological materials. For our business practices, we strictly comply with the following policies:

- We are committed to operating under the highest ethical and legal standards in the acquisition of human and animal tissues used for the preparation of primary cells and other biological materials.
- We warrant that human tissues have been obtained in compliance with local, state, and federal laws and regulations.
- We strictly adhere to the guidelines for human tissue collection and distribution according to established protocols.
- Human tissue used for the isolation of primary cells is derived from donors who have signed informed consent by the donor themselves or an authorized agent acting on the donor's behalf.
- We protect the privacy and autonomy of all donors.
- We believe that human actions have social, economic, and environmental impact, and that they should promote good and avoid harm.
- We believe in the integrity of our activities in the field of biotechnology and that they should be conducted honestly, truthfully, lawfully, impartially, competently, and with considered regard for transparency of process.
- We recognize that individuals and institutions are inherently responsible for their actions and the justification, purpose, and consequences of any action should be taken into account when it is determined. We rely on the protocols, policies, and procedures noted here to provide guidance for the application of ethical behavior in our work as members of the life science society.
- True to our mission, ScienCell provides our products to serve the needs of the life science research community around the world in their pursuit of curing disease and improving life.
