# Supplementary figures and images for: Interaction between mesenchymal stem cells and endothelial cells restores endothelial permeability via paracrine hepatocyte growth factor in vitro
Source: Stem Cell Res Ther. 2015 Mar 24;6(1):44. doi: 10.1186/s13287-015-0025-1 (PMC4431320; doi:10.1186/s13287-015-0025-1)

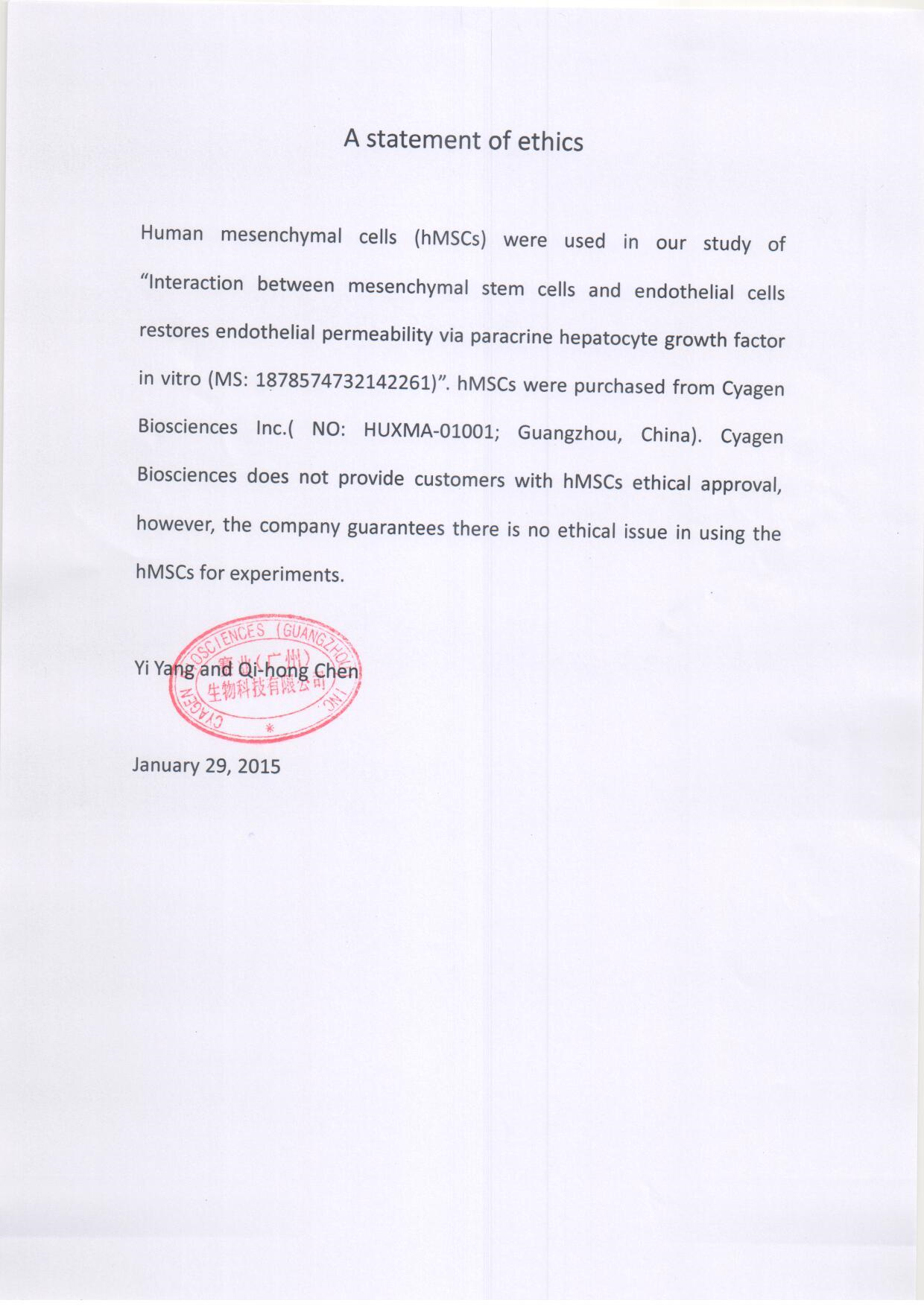

Supplement: Additional file 2: — Ethical approval for human pulmonary microvascular endothelial cell (HPMEC) use. An ethical approval was provided to guarantee there was no ethical issue in using the HPMECs for experiments. [file 13287_2015_25_MOESM2_ESM.png]
